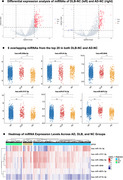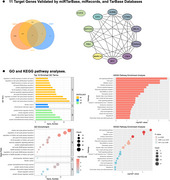# Exploration Shared Pathological Mechanisms in Dementia with Lewy Bodies and Alzheimer's Disease Based on Serum microRNA Expression Profiling

**DOI:** 10.1002/alz70856_102858

**Published:** 2025-12-26

**Authors:** Xin Ma, Weiping Zhang, Chun Tang, Huali Wang

**Affiliations:** ^1^ Peking University Institute of Mental Health (Sixth Hospital), Beijing, Beijing, China; ^2^ Zhejiang University School of Medicine, Hangzhou, Zhejiang Province, China; ^3^ Peking University, Beijing, Beijing, China; ^4^ Dementia Care and Research Center, Peking University Institute of Mental Health (Sixth Hospital), Beijing, China; ^5^ NHC Key Laboratory for Mental Disorders, Beijing, Beijing, China; ^6^ National Clinical Research Center for Mental Disorders, Beijing, China

## Abstract

**Background:**

Dementia with Lewy Bodies (DLB) and Alzheimer's Disease (AD) are two prominent neurodegenerative disorders that exhibit overlapping clinical and pathological features. This study was amid to investigate the shared pathological mechanisms associated with both DLB and AD through the analysis of serum microRNA (miRNA) expression profiles.

**Method:**

We utilized the GSE120584 dataset from the GEO database to identify differentially expressed miRNAs in serum samples from patients with DLB and AD, compared to normal controls (NC), using the *limma* package in R. Then, differentially expressed miRNAs were used to predict target genes with the *multimiR* package. The *Cytoscape* software was used to identified the key hub genes. Gene Ontology (GO) and Kyoto Encyclopedia of Genes and Genomes (KEGG) pathway analyses were conducted on these target genes respectively.

**Result:**

The study included 1,021 AD patients, 169 DLB patients, and 288 NC. Differential expression analysis revealed 6 miRNAs in both DLB *versus* NC and AD *versus* NC: a total of 5 downregulated miRNAs (hsa‐miR‐6875‐3p, hsa‐miR‐6716‐3p, hsa‐miR‐4747‐3p, hsa‐miR‐3646, hsa‐miR‐208a‐5p) and one upregulated miRNA (hsa‐miR‐24‐3p). Key hub gene analysis identified MYC, BRCA1, CDKN2A, and CDK4 as common critical target genes for both DLB and AD. Then, GO and KEGG pathway analysis suggested that these miRNAs may contribute to the overlap pathological mechanisms of DLB and AD by regulating common biological processes and signaling pathways, including cellular senescence, p53 signaling pathway, FoxO signaling pathway, and HIF‐1 signaling pathway.

**Conclusion:**

This study identified six differential expressed serum miRNAs in patients both with DLB and AD which may target at downstream genes and regulate potential signaling pathways, contributing to the overlapping clinical and pathological features between DLB and AD.